# Organization, functions, and mechanisms of the BBSome in development, ciliopathies, and beyond

**DOI:** 10.7554/eLife.87623

**Published:** 2023-07-19

**Authors:** Xiaoyu Tian, Huijie Zhao, Jun Zhou

**Affiliations:** 1 Center for Cell Structure and Function, Shandong Provincial Key Laboratory of Animal Resistance Biology, Collaborative Innovation Center of Cell Biology in Universities of Shandong, College of Life Sciences, Shandong Normal University Jinan China; 2 State Key Laboratory of Medicinal Chemical Biology, Haihe Laboratory of Cell Ecosystem, College of Life Sciences, Nankai University Tianjin China; Fox Chase Cancer Center United States; Fox Chase Cancer Center United States

**Keywords:** Cilia, BBSome, cytoskeleton, ciliopathy

## Abstract

The BBSome is an octameric protein complex that regulates ciliary transport and signaling. Mutations in BBSome subunits are closely associated with ciliary defects and lead to ciliopathies, notably Bardet-Biedl syndrome. Over the past few years, there has been significant progress in elucidating the molecular organization and functions of the BBSome complex. An improved understanding of BBSome-mediated biological events and molecular mechanisms is expected to help advance the development of diagnostic and therapeutic approaches for BBSome-related diseases. Here, we review the current literature on the structural assembly, transport regulation, and molecular functions of the BBSome, emphasizing its roles in cilium-related processes. We also provide perspectives on the pathological role of the BBSome in ciliopathies as well as how these can be exploited for therapeutic benefit.

## Introduction

Bardet-Biedl syndrome (BBS) is a rare, pleiotropic inherited autosomal recessive disorder characterized by retinal dystrophy, obesity, polydactyly, structural and functional renal abnormalities, and learning disabilities (Zhao and Rahmouni, 2022; Tsang et al., 2018). Individuals with BBS can have other abnormalities including craniofacial dysmorphisms, hearing loss, and neurodevelopmental abnormalities, which were widely used to diagnose BBS before the widespread adoption of molecular genetic testing (Zhao and Rahmouni, 2022; Tsang et al., 2018). As a rare disease, BBS affects approximately 1:13,500–1:100,000 people around the world (Chandra et al., 2022). Since there is currently no cure for the multisystem dysfunction seen in BBS, patients with BBS can only benefit from early multidisciplinary intervention with physical therapy, visual services, and support with gastrointestinal control. Thus, identifying the underlying pathobiology could significantly facilitate the clinical diagnosis and management of BBS.

To date, 26 genes have been reported to be related to BBS, 8 of which are involved in the formation of the BBSome, a stable octameric complex (BBS1, BBS2, BBS4, BBS5, BBS7, BBS8/TTC8, BBS9, and BBS18/BBIP1) that shares common structural elements with canonical coat complexes (Jin et al., 2010; Loktev et al., 2008; Nachury et al., 2007). Interestingly, most BBS proteins localize to the basal body or the primary cilium, a microtubule-based projection on the cell surface containing an axoneme that extends from the mother centriole (the basal body) and a membrane sheath, highlighting that cilia are the major sites of BBSome action (Prasai et al., 2020; Nishimura et al., 2019; Nachury and Mick, 2019; Anvarian et al., 2019; Mill et al., 2023).

Primary cilia specialize in propagating signaling cascades, benefitting from their small volume relative to the cell body and enrichment in signaling molecules, such as G-protein-coupled receptors (GPCRs), hedgehog, Wnt, Notch, Hippo, platelet-derived growth factor (PDGF), mammalian target of rapamycin (mTOR), and transforming growth factor-β (TGF-β) (Wachten and Mick, 2021). Given their central role in cell signaling, ciliary defects lead to a broad spectrum of disorders with overlapping phenotypes, called ciliopathies (Nishimura et al., 2019). Molecular transport inside the cilium relies on the intraflagellar transport (IFT) machinery formed from two stable complexes, IFT-A and IFT-B, to bridge ciliary cargoes with the motors responsible for bidirectional transport along the microtubule axoneme. In the cilium, the BBSome acts as an adaptor between the IFT machinery and membrane proteins, making it essential for establishing specific ciliary compartmentalization of signaling molecules (Lechtreck, 2022). Disruption of the BBSome leads to ciliary mislocalization of membrane receptors, abnormal signal transduction, and thus BBS (a subgroup of ciliopathies).

Although structural and genetic studies have significantly broadened our understanding of the BBSome and BBS, details of the BBSome-related molecular mechanisms underpinning the pathobiology of BBS remain unclear. Here, we review recent progress in the organization, functions, and molecular mechanisms of the BBSome in development and ciliopathies, and in doing so provide insights to inform future studies and therapeutic development.

## Physiological and pathological roles of the BBSome in diseases

### Bardet-Biedl syndrome

In 1866, two ophthalmologists, Laurence and Moon, reported the cases of patients with familial blindness, obesity, cognitive deficits, and spastic paraparesis (Laurence and Moon, 1995). Later descriptions of additional symptoms by Bardet and Biedl resulted in the description of ‘Laurence-Moon-Bardet-Biedl syndrome’. Currently, however, Laurence-Moon and Bardet-Biedl syndromes are recognized as separate entities on the same disease spectrum. Diagnosis is based on the presence of four major symptoms (retinal degeneration, postaxial polydactyly, truncal obesity, cognitive impairment, hypogonadism, and renal anomalies) or three major symptoms plus two minor symptoms (speech delay, developmental delay, diabetes mellitus, dental anomalies, congenital heart disease, brachydactyly/syndactyly, ataxia/poor coordination, anosmia/hyposmia) (Caba et al., 2022). Over 26 gene loci have now been attributed to BBS symptoms, the functions of which can be subtyped into three categories: the BBSome, BBSome assembly chaperonins, and other IFT and ciliary proteins (Caba et al., 2022). Mutations in any BBSome subunit can cause BBS, suggesting that every subunit of the BBSome is essential for complete BBSome function.

Corresponding to the symptoms of BBS, the BBSome is necessary for the developmental process and homeostasis of various organs, including the structural organization of photoreceptor outer segments (Hsu et al., 2017; Mayer et al., 2022), neurological functions (Wang et al., 2021; Pak et al., 2021), adipose tissue maturation (Marion et al., 2012a), and renal development (Marchese et al., 2020). The loss of BBS genes has been reported to result in the ciliary dislocation of hedgehog signaling components, which may account for the embryonic developmental defects and polydactyly phenotypes in BBS (Eguether et al., 2014; Hey et al., 2021; Seo et al., 2011; Zhang et al., 2012a). In the eye, rhodopsin localization defects in the inner and outer segments of photoreceptors, and consequent photoreceptor apoptosis are considered causes of the retinal degeneration seen in several animal models of BBS (Nishimura et al., 2004; Davis et al., 2007; Abd-El-Barr et al., 2007; Jiang et al., 2016). Recent studies also emphasize the importance of non-outer segment protein accumulation (Datta et al., 2015; Song et al., 2020; Dilan et al., 2018) and lipid homeostasis (Masek et al., 2022) in the outer segments, which also contribute to BBS-related retinal degeneration. The cognitive defects in BBS can be explained by abnormalities in neurogenesis (Pak et al., 2021) and mislocalization of ciliary receptors in neuronal cilia (Berbari et al., 2008a; Berbari et al., 2008b; Domire et al., 2011). Specifically, the mislocalization of some receptors, such as neuropeptide Y receptor Y2 (NPY2R), serotonin 5-hydroxytryptamine (HT)_2C_ receptor (5-HT_2C_R), and leptin receptor, in the hypothalamus may lead to hyperphagia and obesity (Guo et al., 2019; Guo et al., 2016; Seo et al., 2009). In the periphery, BBS proteins such as BBS4 (Anosov and Birk, 2019; Aksanov et al., 2014), BBS10, and BBS12 (Marion et al., 2009) also regulate adipogenesis. The reason for the renal pathophysiology seen in BBS is less well understood but can be partially attributed to the incorrect ciliary targeting of some transmembrane proteins, such as polycystins 1 and 2 (Su et al., 2014).

Our understanding of the pathogenic role played by the BBSome in BBS patients has benefitted from several animal models including mice, zebrafish, and *Caenorhabditis elegans*, which recapitulate some of the human phenotypes (Song et al., 2020; Kretschmer et al., 2019; Bentley-Ford et al., 2022). For example, the zebrafish is a well-established animal model used to investigate the role of the BBSome in photoreceptor development (Masek et al., 2022). Indeed, recent use of the BBS4 mouse model established a link between immune/hematopoietic defects and BBS (Tsyklauri et al., 2021). However, animal models do not always precisely reproduce the clinical manifestations due to physiological differences between different species. The application of patient-derived cellular models, especially induced pluripotent stem cells (iPSCs) that can be reprogrammed and differentiated into multifunctional tissues, represents a technological solution for BBS disease modeling and *in vitro* assessment of personalized therapies (Wang et al., 2021; Hey et al., 2021; Hey et al., 2019). For example, analysis of the BBS1-M390R mutation model of iPSC-derived hypothalamic arcuate-like neurons revealed downregulation of insulin and leptin signaling pathways, which may contribute to energy homeostasis regulation by the BBSome in neurons (Wang et al., 2021). Improving our understanding of the dynamics of BBS pathology using these disease models will shed further light on the mechanisms of disease onset in humans and help in the discovery and optimization of new treatment strategies.

### Ciliopathies

Besides BBS, nephronophthisis (NPHP), Joubert (JBTS), Meckel-Gruber (MKS), and oral-facial digital (OFD) syndromes are all diseases caused by motile and non-motile primary cilia dysfunction and are collectively known as ciliopathies. These diseases often share common signatures including retinal degeneration, renal or liver dysfunction, polydactyly, brain anomalies, and cognitive impairment. Ciliopathies are genetically complicated disorders, as single gene variants can associate with different ciliopathies (although particular genes are most commonly associated with a specific ciliopathy). Individual ciliopathy patients can also contain variants in multiple genes, which may partially explain the observed symptom diversity as a result of genetic interactions (Muller et al., 2010; Beales et al., 2003; Badano et al., 2003b).

As suggested by their names, BBSome proteins are closely associated with the ciliopathy BBS. However, variants in BBSome proteins have also been identified in some additional ciliopathies and non-ciliated diseases including MKS and the non-syndromic retinitis pigmentosa (Jenkins and Hernandez-Hernandez, 2015). For example, variants in BBS genes (*BBS2*, *BBS4*, and *BBS6*) result in MKS-like phenotypes (Karmous-Benailly et al., 2005), while pathogenic variants in MKS-associated genes (*MKS1*/*BBS13*, centrosomal proteins 290 (*CEP290*/*MKS4*/*BBS14*)) cause clinical phenotypes of BBS (Leitch et al., 2008).

The transition zone is a mutation hub for several ciliopathies including MKS, and genetic interactions between the BBSome and the transition zone complexes cooperatively support ciliary functions (Gonçalves and Pelletier, 2017; Yee et al., 2015). As observed in *Trypanosoma brucei*, the BBSome localizes to a more distal portion of the transition zone than the MKS complex and is seldom observed to overlap with MKS proteins, probably due to rapid shuttling across the transition zone (Dean et al., 2016). Although there are no obvious physical interactions between BBSomes and MKS1, genetic interactions are observed (Leitch et al., 2008). Studies in zebrafish show that partially compromised MKS1 function aggravates the severity of loss of BBS protein function, and *Mks1/Bbs4* double-mutant mouse embryos exhibit exacerbated defects in hedgehog-dependent patterning compared with either mutant alone (Leitch et al., 2008; Goetz et al., 2017). In mice, double mutants in *Bbs1* and another MKS complex component, tectonic family member 1 (*Tctn1*), form cilia at a substantially lower frequency than *Tctn1* single mutants (Yee et al., 2015). Further, binding of BBSomes to other transition zone proteins, such as nephrocystin 5 (NPHP5) and CEP290, has also been reported (Barbelanne et al., 2015). Additional loss of *Bbs4* alleles in C*ep290^rd16^*-mutant (a hypomorphic allele that contains an internal in-frame deletion of 1599–1897 amino acids from the CEP290 protein) mice increases their body weight and accelerates photoreceptor degeneration compared with *Cep290^rd16^* single homozygous mutant mice, suggesting a modifying role for the BBSome in CEP290 ciliopathy phenotypes (Zhang et al., 2014). Genetic interactions have also been observed for *BBS5* and *NPHP1/4* (Bentley-Ford et al., 2022; Yee et al., 2015). Thus, the transition zone complex genetically interacts with the BBSome to maintain cilia homeostasis and modify the phenotypic consequences of each other, which may account for the wide clinical and phenotypic spectra observed in BBSome mutants.

## Structure and formation of the BBSome

### BBS proteins and the BBSome

Discovery of the BBSome was driven by the identification of the genes associated with BBS phenotypes. The first identified BBS gene, *BBS6*, which is similar to group II chaperonins, was discovered at the beginning of the 21st century by positional cloning (Katsanis et al., 2000; Slavotinek et al., 2000). Subsequently, other BBS genes including *BBS1*, *2*, and *4* were cloned using similar methods (Mykytyn et al., 2002; Mykytyn et al., 2001; Nishimura et al., 2001). However, given that these proteins did not share any similarities with other characterized proteins at that time, their function remained elusive.

Nevertheless, other BBS genes were soon identified by screening similar sequences of the cloned BBS genes with the help of bioinformatic comparisons. For example, searching the conceptual translation of the human subset of the EST database with the human and zebrafish BBS2 peptide sequence defined a novel BBS locus corresponding to BBS7 (Badano et al., 2003a). However, it was not until the discovery of BBS8, with its region of similarity to the BBS4 protein sequence and localization to the basal body of ciliated cells, that BBS proteins were hypothesized to be related to ciliary function (Ansley et al., 2003). Notably, patients with *BBS8* variants possess defects in left-right axis determination, which is reminiscent of the nodal cilia dysfunction phenotype (Ansley et al., 2003). The attempt to demonstrate the ciliary role of BBS8 paved the way for the study of ciliary function in BBS. Indeed, most BBS proteins now have established roles in BBSome- or cilia-related functions, and the major functions of the twenty-six BBS proteins are listed in Table 1.

**Table 1.** Characteristics and functions of *BBS* genes.

No	Gene Symbol	Gene Name	Protein	Functions	References
1	*BBS1*	Bardet-Biedl syndrome 1	Bardet-Biedl syndrome 1 protein	Member of the BBSome complex	Mykytyn et al., 2002
2	*BBS2*	Bardet-Biedl syndrome 2	Bardet-Biedl syndrome 2 protein	Member of the BBSome complex	Nishimura et al., 2001
3	*ARL6*	ADP ribosylation factor like GTPase 6	ADP-ribosylation factor-like protein 6	Small GTPase; Facilitate BBSome assembly and recruitment to the cilium	Chiang et al., 2004; Fan et al., 2004
4	*BBS4*	Bardet-Biedl syndrome 4	Bardet-Biedl syndrome 4 protein	Member of the BBSome complex	Mykytyn et al., 2001
5	*BBS5*	Bardet-Biedl syndrome 5	Bardet-Biedl syndrome 5 protein	Member of the BBSome complex	Woods et al., 1999; Young et al., 1999
6	*MKKS*	MKKS centrosomal shuttling protein	McKusick-Kaufman/Bardet-Biedl syndromes putative chaperonin	Chaperonin protein for BBSome complex assembly	Katsanis et al., 2000; Slavotinek et al., 2000
7	*BBS7*	Bardet-Biedl syndrome 7	Bardet-Biedl syndrome 7 protein	Member of the BBSome complex	Badano et al., 2003a
8	*TTC8*	Tetratricopeptide repeat domain 8	Tetratricopeptide repeat protein 8	Member of the BBSome complex	Ansley et al., 2003
9	*BBS9*	Bardet-Biedl syndrome 9	Protein PTHB1	Member of the BBSome complex	Nishimura et al., 2005
10	*BBS10*	Bardet-Biedl syndrome 10	Bardet-Biedl syndrome 10 protein	Chaperonin protein for BBSome complex assembly	Stoetzel et al., 2006
11	*TRIM32*	Tripartite motif containing 32	E3 ubiquitin protein ligase TRIM32	E3 ubiquitin ligase	Chiang et al., 2006
12	*BBS12*	Bardet-Biedl syndrome 12	Bardet-Biedl syndrome 12 protein	Chaperonin protein for BBSome complex assembly	Stoetzel et al., 2007
13	*MKS1*	MKS transition zone complex subunit 1	Meckel syndrome type 1 protein	Transition zone component; Regulates ciliary trafficking	Leitch et al., 2008
14	*CEP290*	Centrosomal protein 290	Centrosomal protein of 290 kDa (Cep290)	Transition zone component; Regulates ciliary entry	Leitch et al., 2008
15	*WDPCP*	WD repeat containing planar cell polarity effector	WD repeat containing and planar cell polarity effector protein fritz homolog (hFRTZ)	Component of the CPLANE (ciliogenesis and planar polarity effectors) complex; regulates ciliogenesis	Kim et al., 2010
16	*SDCCAG8*	SHH signaling and ciliogenesis regulator SDCCAG8	Serologically defined colon cancer antigen 8	Regulates ciliogenesis and Hedgehog signaling pathway	Otto et al., 2010
17	*LZTFL1*	Leucine zipper transcription factor like 1	Leucine zipper transcription factor-like protein 1	Regulates the BBSome trafficking	Marion et al., 2012b
18	*BBIP1*	BBSome interacting protein 1	BBSome-interacting protein 1	Member of the BBSome complex	Loktev et al., 2008
19	*IFT27*	Intraflagellar transport 27	Intraflagellar transport protein 27 homolog	IFT-B complex component; Required for ciliary trafficking	Aldahmesh et al., 2014
20	*IFT172*	Intraflagellar transport 172	Intraflagellar transport protein 172 homolog	IFT-B complex component	Bujakowska et al., 2015
21	*CFAP418*	Cilia And Flagella Associated Protein 418	Cilia- and flagella-associated protein 418	A ciliary protein of unknown function	Khan et al., 2016; Heon et al., 2016
22	*IFT74*	Intraflagellar transport 74	Intraflagellar transport protein 74 homolog	IFT-B complex component	Lindstrand et al., 2016
23	*NPHP1*	Nephrocystin 1	Nephrocystin-1	Transition zone component	Lindstrand et al., 2014
24	*SCAPER*	S-phase cyclin A associated protein in the ER	S phase cyclin A-associated protein in the endoplasmic reticulum (S phase cyclin A-associated protein in the ER)	Regulates ciliary dynamics	Wormser et al., 2019
25	*CCDC28B*	Coiled-coil domain containing 28B	Coiled-coil domain-containing protein 28B	Centrosomal protein that regulates ciliogenesis	Badano et al., 2006
26	*SCLT1*	Sodium channel and clathrin linker 1	Sodium channel and clathrin linker 1	Distal appendage component that regulates ciliogenesis	Morisada et al., 2020

In 2007, Nachury et al. applied localization and tandem affinity purification (LAP) to BBS4 in hTERT RPE-1 (RPE-1) cells and pulled down six other BBS proteins (BBS1, BBS2, BBS5, BBS7, BBS8, and BBS9) in a stoichiometric ratio with BBS4. This indicated that these seven BBS proteins form a stable complex, which they named the BBSome (Nachury et al., 2007). Another 10 kDa BBSome subunit, BBS18, was identified one-year later (Loktev et al., 2008), completing the identification of the entire octameric protein complex. Later, another subset of BBS proteins (BBS6, BBS10, and BBS12) were demonstrated to mediate BBSome assembly (Seo et al., 2010).

As well as these BBS proteins, some IFT-B complex components (IFT74, IFT172, and IFT27) have also been reported in association with BBS, suggesting that BBS proteins genetically interact with IFT components (Zhou et al., 2022b; Bujakowska et al., 2015; Luo et al., 2021). In addition, clinical case reports of different BBS families identified other BBS proteins, some of which are associated with additional ciliopathies. For example, variants in the transition zone protein *MKS1* result in MKS and BBS, while *CEP290* variants are associated with NPHP, JBTS, and BBS (Leitch et al., 2008; Coppieters et al., 2010). These findings indicate that variants in genes encoding ciliary proteins, especially transition zone and IFT proteins, share a wide range of phenotypes, and more BBS proteins will potentially be identified from those proteins.

### Molecular structure and assembly of the BBSome

#### Assembly of the BBSome

Consisting of eight subunits, BBSome assembly is thought to proceed sequentially, with all BBSome subunits interdependent to create stability (Prasai et al., 2020; Zhang et al., 2012b). In addition to the core BBSome subunits, several other BBS proteins are important for BBSome assembly, including chaperonin-like BBS proteins and small GTPases.

Three non-core BBS proteins, BBS6, BBS10, and BBS12, have sequence homology with the CCT/TRiC family of group II chaperonins (Katsanis et al., 2000; Stoetzel et al., 2006; Stoetzel et al., 2007). Chaperone proteins represent a ubiquitous and essential protein family involved in many fundamental biological processes including correct protein folding and refolding, transport, and quality control (Gupta et al., 2022). Of these, CCT family chaperonins form a hetero-oligomeric complex consisting of two stacked rings composed of eight radially arranged subunits (CCT1–8) that mediate protein folding in an ATP-dependent manner (Ghozlan et al., 2022). Variants in *BBS6*, *BBS10*, or *BBS12* typically cause more severe phenotypes than those of the core BBSome components (Álvarez-Satta et al., 2017). Furthermore, variants in *BBS6*, *BBS10*, or *BBS12* together account for a large proportion of BBS cases (over 30%, compared with the 28% for BBS1), indicating they might function at a relatively early step of BBSome formation (Niederlova et al., 2019). BBS6, BBS10, and BBS12 form a higher order complex with six CCT chaperonin proteins (CCT1, CCT2, CCT3, CCT4, CCT5, and CCT8), and the BBSome fails to assemble when this complex is absent (Seo et al., 2010). Considering that BBS6 and BBS12 do not contain an ATP-binding motif and the ATPase activity of BBS10 is not validated either, the protein folding activity of the complex is accomplished through the incorporated CCT chaperonins (Katsanis et al., 2000; Stoetzel et al., 2006; Stoetzel et al., 2007). In fact, BBS6, BBS10, and BBS12 act as the substrate-binding unit of the CCT chaperonins responsible for mediating the association between CCT chaperonins and BBS7 to stabilize BBS7 and facilitate its association with BBS2, before their transition to other BBSome subunits (Seo et al., 2010). BBS10 is not a structural component of the BBS-chaperonin complex, but rather regulates its formation (Zhang et al., 2012b).

**Figure 1.**
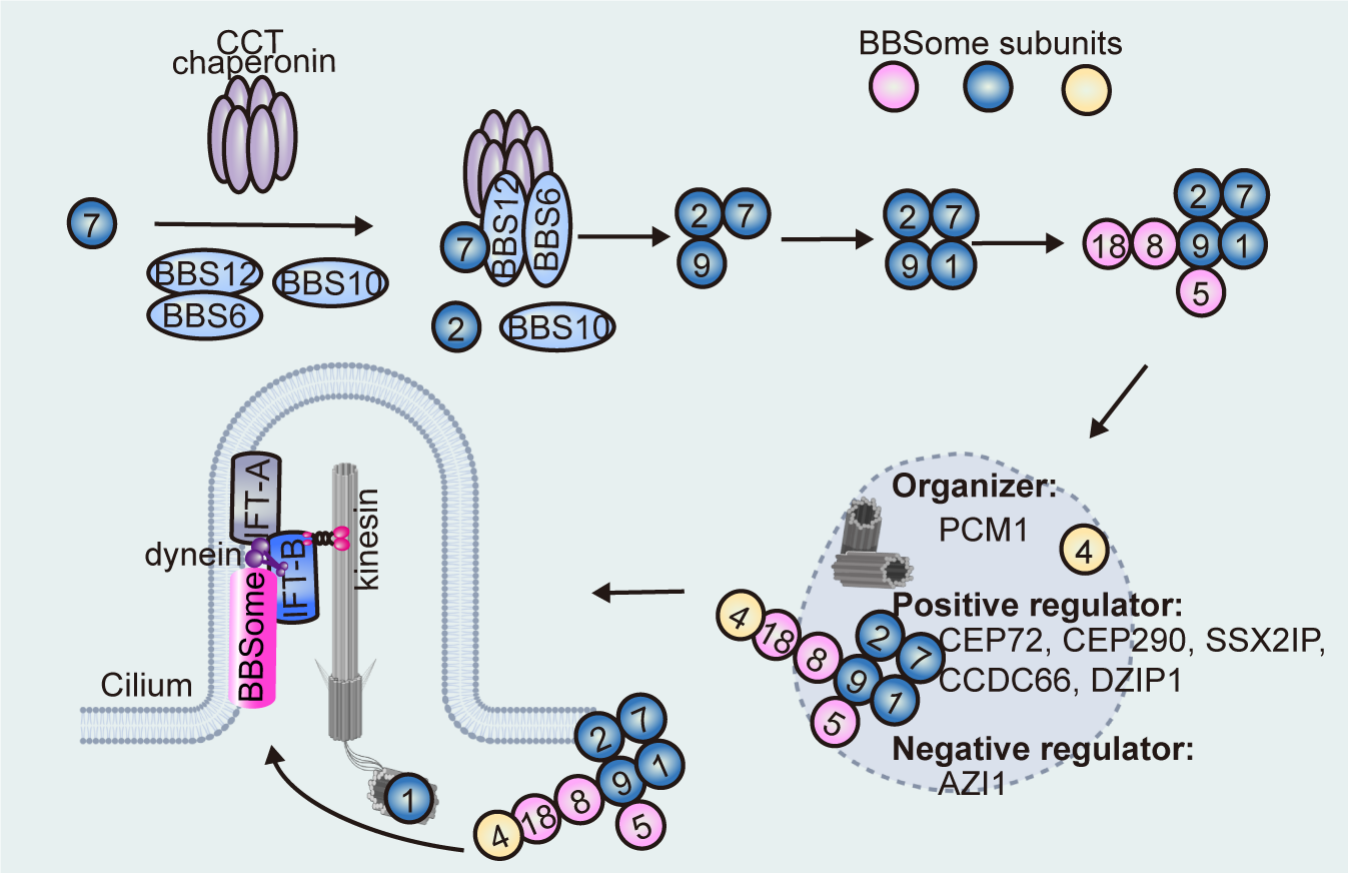
Sequential assembly model of the BBSome. The CCT chaperonin complex and BBS6/10/12 stabilize BBS7 to form the BBS-chaperonin complex, which recruits BBS2 for BBS7 binding. BBS2 directly interacts with BBS9 and BBS7 to form a ternary core complex before subsequent recruitment of other BBSome subunits. BBS4, which localizes on the centriolar satellites, is presumably the last subunit to be incorporated into the BBSome, whereas the basal body-localized BBS1 facilitates transfer of the BBSome into the cilium. BBSome transfer from centriolar satellites to the basal body (and then to the cilium) is regulated by centriolar satellite proteins (e.g. CEP72, CEP290, SSX2IP, CCDC66, DZIP1, and AZI1).

By exploiting positional mutagenesis and null alleles of BBS proteins to disrupt BBSome assembly, Zhang et al. (Zhang et al., 2012b) characterized BBSome assembly intermediates and the intrinsic protein-protein interactions between BBSome proteins. They identified BBS2-BBS7-BBS9 as an important intermediate complex during the initiation of BBSome assembly, which is coordinated by the release of BBS7 from the BBS-chaperonin complex (Figure 1; Zhang et al., 2012b). BBS2 and BBS7 form a tight dimer brought together by chaperonins, followed by association of BBS9 through binding with BBS2 to form the BBS2-BBS7-BBS9 core. Then, BBS1, BBS5, and BBS8 are incorporated independently into the BBSome by directly interacting with BBS9, while BBS4 is the final subunit added to the BBSome (Zhang et al., 2012b). The protein interaction network revealed by the visible immunoprecipitation (VIP) assay consistently identifies BBS9 as the hub of the BBSome, which organizes a core subcomplex consisting of BBS1, BBS2, BBS7, and BBS9 (Katoh et al., 2015). At the periphery, BBS18 and BBS8 serve as a linker between BBS4 and BBS9, while BBS5 localizes to the periphery of the core complex to bind the membrane (Katoh et al., 2015). Interestingly, some recent studies argue essential roles for BBS2 and BBS7 in initiating BBSome assembly, as *Drosophila melanogaster* does not possess these subunits and their absence leads to a more stable BBSome subcomplex during purification from insect cells (Klink et al., 2017). Of note, CCT chaperonins are also not conserved outside vertebrates, probably because there is no need to incorporate BBS2/7 into the BBSome in *D. melanogaster*, while alternative modes of BBSome assembly must exist as BBSome components are conserved in other invertebrates and protists (such as in *C. elegans* and *Chlamydomonas reinhardtii*). The mechanisms underlying BBSome assembly clearly still require further clarification.

Immunofluorescence-based techniques in living cells have revealed that the BBSome is in fact readily assembled in non-ciliated cells and regulated in a spatiotemporal manner (Prasai et al., 2020). In this context, BBS1 is thought to be the last subunit incorporated into the BBSome, or the complex undergoes a conformational change to complete the translocation of the pre-BBSome from the centriolar satellites to the ciliary base (Prasai et al., 2020). It has been proposed that the BBSome is pre-assembled and recruited to the centriolar satellites by BBS4 (Figure 1), which was the first BBSome component reported to localize to centriolar satellites in non-ciliated cells (Prasai et al., 2020; Kim et al., 2004). In fact, every BBSome component accumulates at centriolar satellites when BBS1 is depleted, which is not observed in *BBS1/BBS4* double-knockout cells (Prasai et al., 2020). Conversely, the centriolar satellite localization of BBS4 is not affected by the deletion of other BBSome components (Zhang et al., 2012b).

Centriolar satellites are important for the efficient localization of centrosomal and ciliary proteins, as they control protein entry to the cilium either through sequestration or trafficking them to or away from the cilium (Tischer et al., 2021). As a scaffold protein for the assembly and maintenance of centriolar satellites, pericentriolar material 1 (PCM1) is essential for proper ciliogenesis and ciliary functions, and *Pcm1* knockout mice display ciliopathy-associated phenotypes such as dwarfism, hydronephrosis, and male infertility (Odabasi et al., 2020; Hall et al., 2023). The N-terminal 380 amino acids of BBS4 interact with the C-terminal of PCM1, and the centriolar satellite localizations of BBS4 and PCM1 are interdependent (Zhang et al., 2012b). BBS4 recruits PCM1 to the centriolar satellites by acting as an adaptor between the p150^glued^ subunit of the dynein-dynactin motor complex and PCM1, while PCM1 serves as a centriolar satellite platform for BBS4 and other centriolar satellite proteins (Zhang et al., 2012b; Kim et al., 2004; Chamling et al., 2014). However, PCM1 depletion does not prevent ciliary localization of BBS4, indicating that it might not be required for the basal body transfer of BBS4 and thus the BBSome, but rather orchestrates or sequesters the BBSome in the centriolar satellite ready for its release to the basal body (Stowe et al., 2012). Consistently, membrane-bound BBSomes pulled down by ARL6/BBS3-GTP do not contain PCM1, implying that PCM1 should be removed before ciliary translocation of the BBSome (Jin et al., 2010). PCM1 also regulates the proper centriolar satellite localizations of several centriolar satellite proteins such as centrosomal proteins 72 (CEP72) and CEP290 (Stowe et al., 2012), SSX family member 2-interacting protein (SSX2IP) (Klinger et al., 2014), and coiled-coil domain-containing 66 (CCDC66) (Conkar et al., 2017), depletion of which impaired the ciliary localization of BBSome. Another centriolar satellite protein, AZI1/CEP131, physically binds the BBSome *via* BBS4 and sequesters the BBSome in the centriolar satellite to prevent its release into the cilium (Chamling et al., 2014), while leucine zipper transcription factor-like 1 (LZTFL1/BBS17) associates with and sequesters the BBSome in the cytoplasm to limit the ciliary entry of the BBSome in mammalian cells (Seo et al., 2011). Cell cycle-dependent regulation has also been reported. For example, it has been reported that DAZ-interacting zinc finger protein 1 (DZIP1) assembles the BBSome-DZIP1-PCM1 complex at centriolar satellites for ciliary targeting in G_0_ phase, while it is phosphorylated at Ser210 by polo-like kinase 1 (PLK1) to disassociate from centriolar satellites and decrease the centriolar satellite association of the BBSome (Zhang et al., 2017). In summary, these regulatory proteins may provide a quality control mechanism that blocks the release of incomplete pre-BBSomes from centriolar satellites (Figure 1). However, no proper centriolar satellites or orthologues of centriolar satellite proteins, such as PCM1 and CEP72, were identified in *C. reinhardtii* (Stowe et al., 2012; Keller et al., 2005), suggesting that the centriolar satellite-dependent assembly and recruitment of BBSome to the cilia base is not conserved between species.

**Figure 2.**
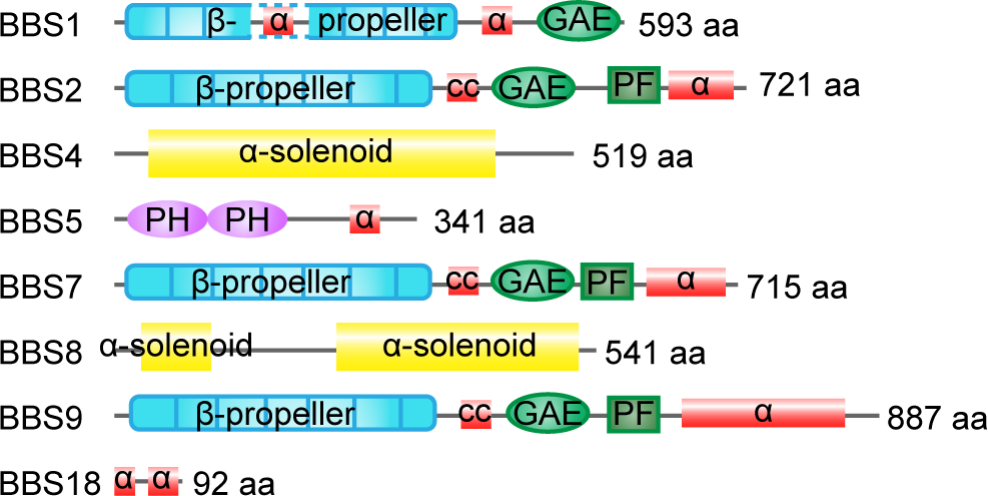
Domain organization of homo BBSome subunits. The number of residues and the domain structures are indicated. α, alpha helices; GAE, gamma-adaptin ear domain; cc, coiled-coil domain; PF, platform domain; PH, pleckstrin-homology domain.

**Figure 3.**
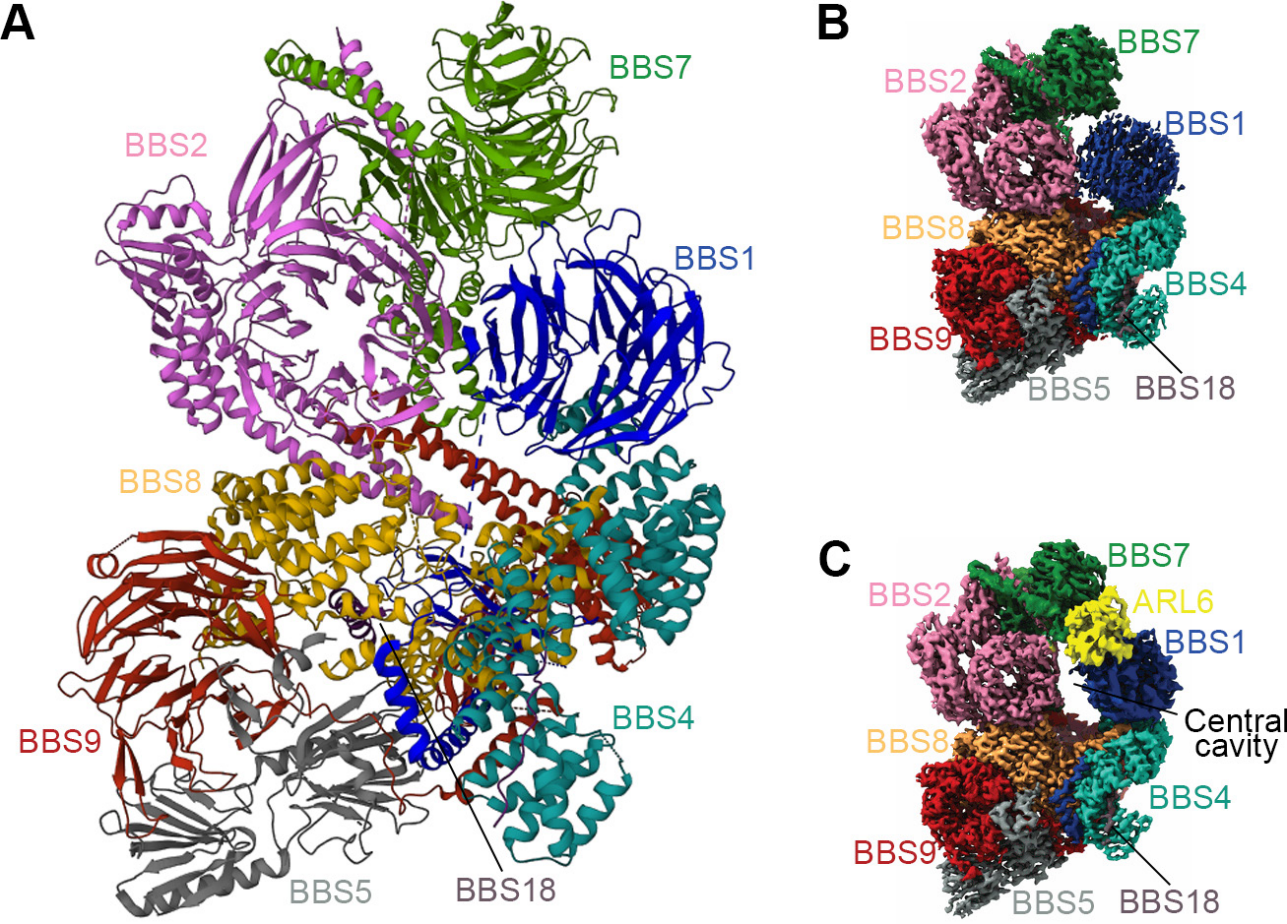
Structure of the mammalian BBSome. (**A**) Atomic models of the eight subunits of the bovine BBSome. (**B**) Cryo-EM structure of the bovine BBSome. (**C**) Cryo-EM structure of the BBSome:ARL6:GTP complex in the same orientations as the map in panel B. Images are adapted from Singh et al., 2020, where it was published under a CC BY 4.0 license.

Structurally, BBS1, BBS2, BBS7, and BBS9 share similar domain architectures including an N-terminal β-propeller domain (Figure 2), suggesting a common evolutionary origin. BBS4 and BBS8 are related through their tetratricopeptide repeat domains, which can fold into solenoids (Figure 2). BBS18 possesses two alpha helices, while BBS5 contains pleckstrin-homology domains (Figure 2). Thanks to the development of biological techniques, including co-immunoprecipitation-based protein-protein interaction assays, GST pull-down, VIP, and the yeast two-hybrid assay, the BBSome interaction network has been narrowed down to specific domains (Woodsmith et al., 2017). For example, the C-terminal 200 amino acids of BBS7 binds the α-helix–rich C-terminal domain of BBS2, with binding regulated by the central coiled-coil domain of BBS2 (Seo et al., 2010; Zhang et al., 2013). BBS9 indirectly interacts with BBS7 by binding the C-terminus of BBS2 (Zhang et al., 2012b; Woodsmith et al., 2017). The C-terminal domain of BBS9 is responsible for binding BBS1 and BBS4, while the N-terminal domain of BBS9 binds BBS8 and BBS5 (Woodsmith et al., 2017). Detailed insights into the BBSome interaction network (Figure 3A) will explain how some of the genetic variations in the BBSome give rise to specific functional defects and lead to diseases.

BBSome assembly is also regulated by the small Arf-like GTPase ARL6, which helps the BBSome to assemble into a coat complex for membrane targeting (Jin et al., 2010; Mourão et al., 2014). BBS1 binds to the switch regions of the GTP site of ARL6, indicating that the BBSome is an effector for ARL6 (Mourão et al., 2014). The overall formation of the BBSome shows a domain architecture similar to vesicle-forming complex proteins such as COPI/COPII and clathrin, and the interaction between the BBSome and ARL6 is reminiscent of the activation of clathrin-AP complexes by ARF1/SAR1 GTPases (Jin et al., 2010). The detailed mechanisms of regulation of the BBSome by ARL6 are discussed further below.

#### Molecular structure of the BBSome

Determining the crystal structure of the BBSome has been a long-standing challenge, as recombinant BBSome complexes are notoriously difficult to purify. Recent analyses based on single-particle cryoelectron microscopy (cryo-EM) and purified BBSomes from the bovine retina or insect cells have advanced our knowledge about BBSome architecture and provided insights into how the BBSome is organized, recruited to the membrane, and binds to cargoes (Chou et al., 2019; Klink et al., 2020).

To better understand the mechanisms of BBSome assembly, a trimeric BBSome subcomplex of BBS2-BBS7-BBS9 was isolated from HEK-293T cells and resolved by electron microscopy (EM) and chemical crosslinking coupled with mass spectrometry (XL-MS) (Ludlam et al., 2019). The overall structure of the reconstructed trimer (23 Å) resembled a flattened triangle, with BBS2 and BBS7 forming a tight dimer through a coiled-coil interaction involving residues 334–363 of BBS2 and residues 340–363 of BBS7 (Ludlam et al., 2019). BBS9 associated with the dimer by interacting with the α-helix domain of BBS2. The N-terminal domain of purified BBS9 (1.8 Å) folded into a seven-bladed β-propeller, a structure potentially involved in protein-protein interactions to hold the BBSome complex together (Knockenhauer and Schwartz, 2015). In slight contrast to the trimeric model, the mid-resolution (4.9 Å) and high-resolution (3.1 Å and 3.4 Å) cryo-EM reconstructions of the whole BBSome purified from cow retina showed that the β-propeller of BBS9 extends away from the BBS2/7 dimer due to interactions with the other BBSome subunits (Chou et al., 2019; Singh et al., 2020; Yang et al., 2020). In this holo complex (Figure 3A and B), the BBSome is arranged in two lobes, a top lobe (BBS2 and BBS7) and a bottom lobe (BBS1, BBS4, BBS5, BBS8, BBS9, and BBS18) connected by the central helical bundle composed of BBS2 and BBS9 (Singh et al., 2020). Consistent with the binary interaction networks, BBS4 and BBS5 localize to the periphery of the complex, in accordance with their dispensable roles in BBSome assembly, while BBS18 functions as a linker between BBS8 and BBS4. In the case of BBS2 and BBS7, their positions on the BBSome are flexible and decrease the resolution of the whole BBSome complex. As these two components are less soluble by themselves, they probably need chaperonins for incorporation into the complex (Seo et al., 2010; Klink et al., 2017; Singh et al., 2020). However, their interweaved binding increases their interdependence, which may dramatically affect BBSome assembly (Zhang et al., 2012b).

As a critical step for BBSome recruitment to the membrane, the crystal structure of ARL6-GTP-BBS1 (3.1–3.5 Å) revealed that ARL6 binds to the N-terminal ß-propeller domain of BBS1 at blades 1 and 7 (Mourão et al., 2014). In the absence of BBS2 and BBS7, the ß-propeller of BBS1 locates at the periphery of the remaining core BBSome complex (3.8 Å), freely accessible to bind to membrane-attached ARL6 (Klink et al., 2020). This allows a positively charged surface patch of the core BBSome to contact the negatively charged membrane. Interestingly, the anchoring of BBS1-ARL6 to the preexisting holo-BBSome crystal structure (4.9 Å) turns out to be problematic, since the BBSome adopts a conformation that is refractory to ARL6-GTP binding (Chou et al., 2019). The BBSome is thought to exist in a closed conformation before undergoing a conformational change to bind ARL6, and the top lobe (BBS2 and BBS7) must open to allow ARL6 to bind (Figure 3B and C). However, the high-resolution structure of the BBSome-ARL6-GTP complex (3.5 Å) shows that the top lobe remains in a closed, downward conformation, even in the presence of ARL6 (Singh et al., 2020). Instead, the ß-propeller of BBS1 swivels in its cradle between BBS4 and BBS7 to open a central cavity for accommodating ARL6 (Singh et al., 2020; Yang et al., 2020). Similar to the crystal structure resolved by the ARL6-GTP-BBS1 ternary complex, the first and last blades of the BBS1 ß-propeller interact with two loops - helices a3 (residues 75–78) and a4 (residues 98–108) - of the GTP-bound ARL6 (Singh et al., 2020). Thus, a convex, positively charged surface defined by the N-terminal of ARL6-GTP and parts of the BBS2 platform, BBS7 ß-propeller, and BBS9 ß-propeller, allows these complexes to associate with the concave membrane (Yang et al., 2020).

To recruit the BBSome to the membrane, it was previously thought that BBS5 is responsible for mediating membrane contact by binding phosphoinositides through pleckstrin homology domains (Nachury et al., 2007). However, it now seems that a sub-complex containing BBS4, 8, 9, and 18 binds to phosphoinositides with similar affinity as the core complex containing BBS1, 4, 5, 8, 9, and 18, indicating that BBS5 is not exclusively responsible for phosphoinositide binding (Klink et al., 2020). For the holo-BBSome complex, the predicted PIP-binding sites of BBS5 are occluded by BBS9 and BBS8 (Yang et al., 2020). Thus, more evidence is needed to verify the lipid-binding function of BBS5.

Upon binding to ARL6-GTP, rotation of the BBS1 ß-propeller and the opening of the cavity lead to the formation of a negatively charged cleft potentially involved in cargo recognition (Singh et al., 2020). This cleft might provide space for its cargoes to shelter. The plasticity of BBS1 in its loosely held cradle may allow it to subtly reorient to optimally contact multiple cargoes. Furthermore, the cleft is close to the BBS7 ß-propeller and coiled-coil domains, which are responsible for binding its cargoes such as Smoothened (Smo) (Yang et al., 2020). Thus, ARL6-GTP binding to the BBSome may modify the BBSome to an orientation optimal for cargo binding.

With only limited information available about the high-resolution structure of the BBSome, the detailed arrangement of BBSome subunits still requires further exploration. A detailed structure of the BBSome will provide insights into the interconnected arrangement of its architecture, provide information about the molecular mechanisms underlying BBSome functions, and help to accurately locate pathogenic mutations that perturb the intermolecular interactions and functions of the protein complex.

## Cellular functions of the BBSome

**Figure 4.**
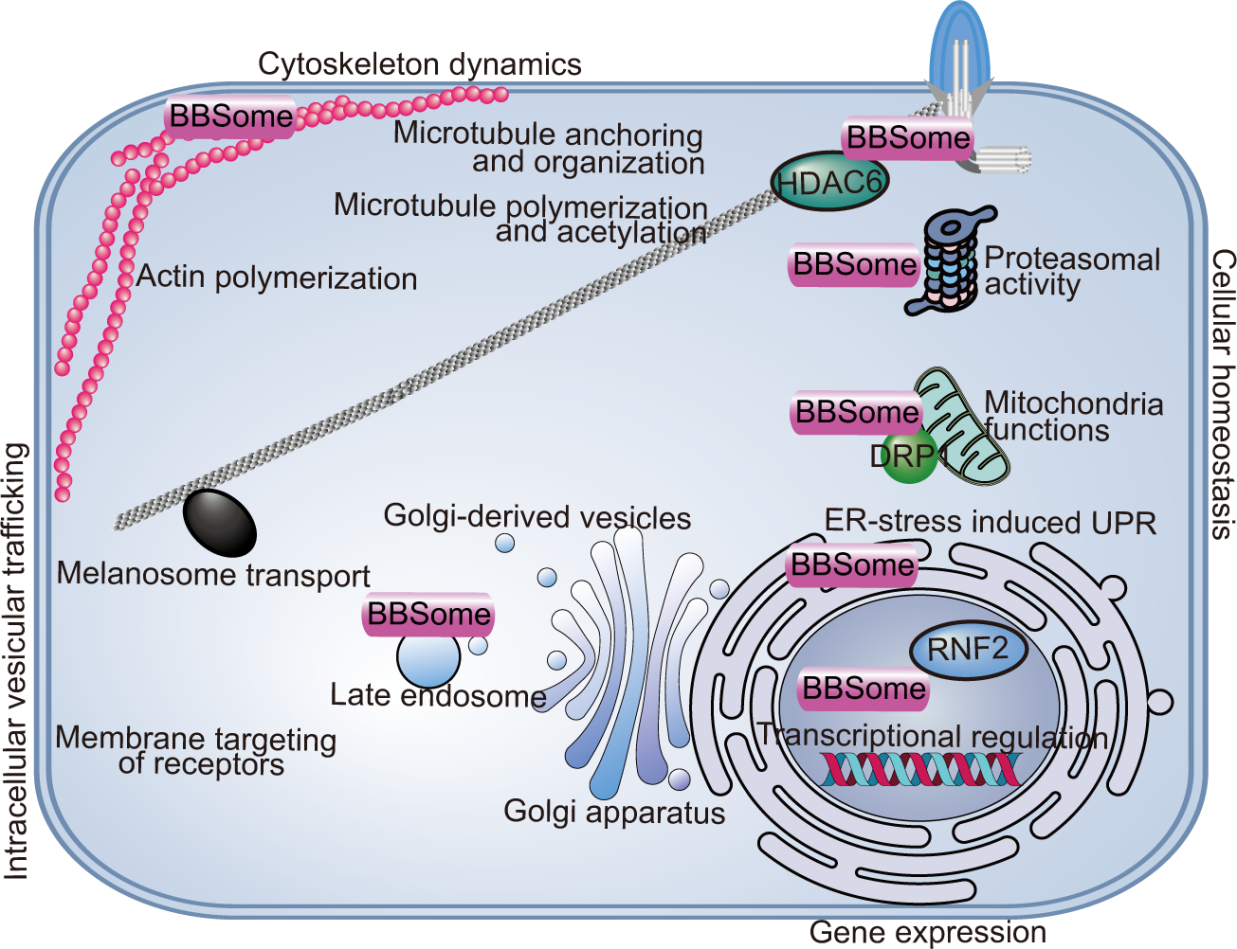
Non-ciliary functions of the BBSome. The BBSome is involved in a wide range of cellular functions including intracellular vesicular transport, cytoskeletal dynamics, gene expression, and cellular and organelle homeostasis. ER, endoplasmic reticulum; UPR, unfolding protein response.

BBSomes have several intracellular functions in addition to their ciliary functions including regulating intracellular vesicular trafficking, cell cytoskeleton dynamics, gene expression, and cellular and organelle homeostasis (Figure 4). This wide range of functions of the BBSome may contribute to the complexity of BBSome-related diseases.

With respect to intracellular vesicular trafficking, the BBSome is required for plasma membrane surface localization of several receptors or membrane proteins including the leptin receptor (Guo et al., 2016), Notch receptor (Leitch et al., 2014), insulin receptor (Starks et al., 2015), 5-HT_2C_R (Guo et al., 2016), and Vangl2 (May Simera et al., 2015). Considering the metabolic and developmental functions of these receptors, their dislocation from the plasma membrane may account for some BBS symptoms, such as obesity, insulin resistance, and planar cell polarity defective phenotypes (Guo et al., 2019; Leitch et al., 2014; Starks et al., 2015; May Simera et al., 2015). In zebrafish, BBSome depletion inhibits the retrograde transport of melanosomes, probably by disrupting intracellular trafficking (Yen et al., 2006). BBSomes colocalize with late endosomes (rather than early endosomes and Golgi apparatus) and are supposed to regulate intracellular trafficking by interacting with components of the secretory pathway (Guo et al., 2019).

Early evidence of compromised cell migration, adhesion, and division in BBS4-deficient cells indicated a role for the BBSome in regulating cytoskeleton dynamics (Hernandez-Hernandez et al., 2013). It has been reported that BBS18 regulates cytoplasmic microtubule polymerization and acetylation by interacting with histone deacetylase 6 (HDAC6) (Loktev et al., 2008). BBS4 depletion or expression of truncated forms of BBS4 similar to those found in some BBS patients leads to defective anchoring of centrosomal microtubules and a failure of cell division, indicating that microtubule disorganization may contribute to BBS phenotypes (Kim et al., 2004). The disruption of cytoplasmic actin polymerization is also reported for BBS4-depleted cells (Hernandez-Hernandez et al., 2013). BBS8 and BBS9 colocalize with focal adhesions, further supporting a role for the BBSome in regulating actin cytoskeleton homeostasis (Hernandez-Hernandez et al., 2013).

BBSomes are predicted to regulate gene expression due to the presence of nuclear localization/export signals, and confirmed nuclear localizations for several BBSome components have been reported (Gascue et al., 2012; Ewerling et al., 2023). In a screen for genes regulating the photoreceptor protein LITE-1 in *C. elegans*, BBSome proteins were selected and shown to regulate LITE-1 stability by transcriptionally regulating DLK-MAPK signaling, providing potential evidence for cilia-independent roles of the BBSome in BBS-related visual loss (Zhang et al., 2022). In another study, BBS7 physically interacted with the polycomb group (PcG) member RNF2 and regulated its downstream targets, most of which are crucial for development and tissue homeostasis (Gascue et al., 2012). Thus, BBSome likely plays roles in transcriptional regulation.

BBSomes also regulate cellular homeostasis through proteasomal degradation of signaling pathway subunits. BBS1, 2, 4, 7, and 8 can all interact with proteasomal subunits, and loss of BBS4 leads to depletion of multiple subunits from the centrosomal proteasome and defective accumulation of proteasome-dependent signaling components (Liu et al., 2014; Gerdes et al., 2007). Furthermore, BBS4 is reported to localize on the endoplasmic reticulum and play a role in the endoplasmic reticulum stress-induced unfolding protein response (UPR) in adipocytes and neuronal cells, potentially influencing early-stage development of the related organs (Anosov and Birk, 2019; Horwitz and Birk, 2021). A recent study also reported a role for the BBSome in mitochondrial functions (Guo et al., 2023). The BBSome regulates mitochondria morphology and function by modulating the phosphorylation and mitochondrial translocation of dynamin-like protein 1 (DRP1), highlighting a role for mitochondrial defects in BBSome-related diseases (Guo et al., 2023). Importantly, rescuing the mitochondrial defects partially reversed neuroanatomical abnormalities, metabolic alterations, and obesity phenotypes in BBSome-deficient mice, highlighting the mitochondrial function of the BBSome in the pathogenesis of BBS (Guo et al., 2023).

Other cell-type-specific functions of the BBSome may include: regulating dendritic spine homeostasis in the postsynaptic density of hippocampal neurons (Haq et al., 2019); regulating astrocyte activity in the brain (Singh et al., 2019); affecting axonal targeting in the olfactory bulb (Tadenev et al., 2011); regulating vascular tone and related physiological processes in endothelial cells (Jiang et al., 2021); impacting photoreceptor synaptogenesis and synaptic contact positioning in the retina (Hsu et al., 2020); regulating aortic stiffness in smooth muscle (Reho et al., 2019); and regulating fibroblast migration through platelet-derived growth factor-AA (PDGF) receptor-α signaling and Cullin-3-mediated control of RhoA (Guo and Rahmouni, 2019). The widespread functions of the BBSome in many different cell types and organs help to explain the pathogenesis of its associated multisystem diseases.

BBS is classified as a ciliopathy, and many clinical aspects of this disorder can be explained by ciliary defects (Ansley et al., 2003). However, some of the pleiotropic symptoms of BBS may also result from the non-ciliary functions of BBS proteins. Note that some of the ciliary and non-ciliary functions of the BBSome may interconnect and overlap, and it is still unclear to what extent the non-ciliary functions of the BBSome contribute to the diverse defects seen in BBS-deficient cells. Thus, it will be important to interpret the mechanisms and functions of the BBSome from a more comprehensive perspective of the entire cell.

## Ciliary regulation of the BBSome

**Figure 5.**
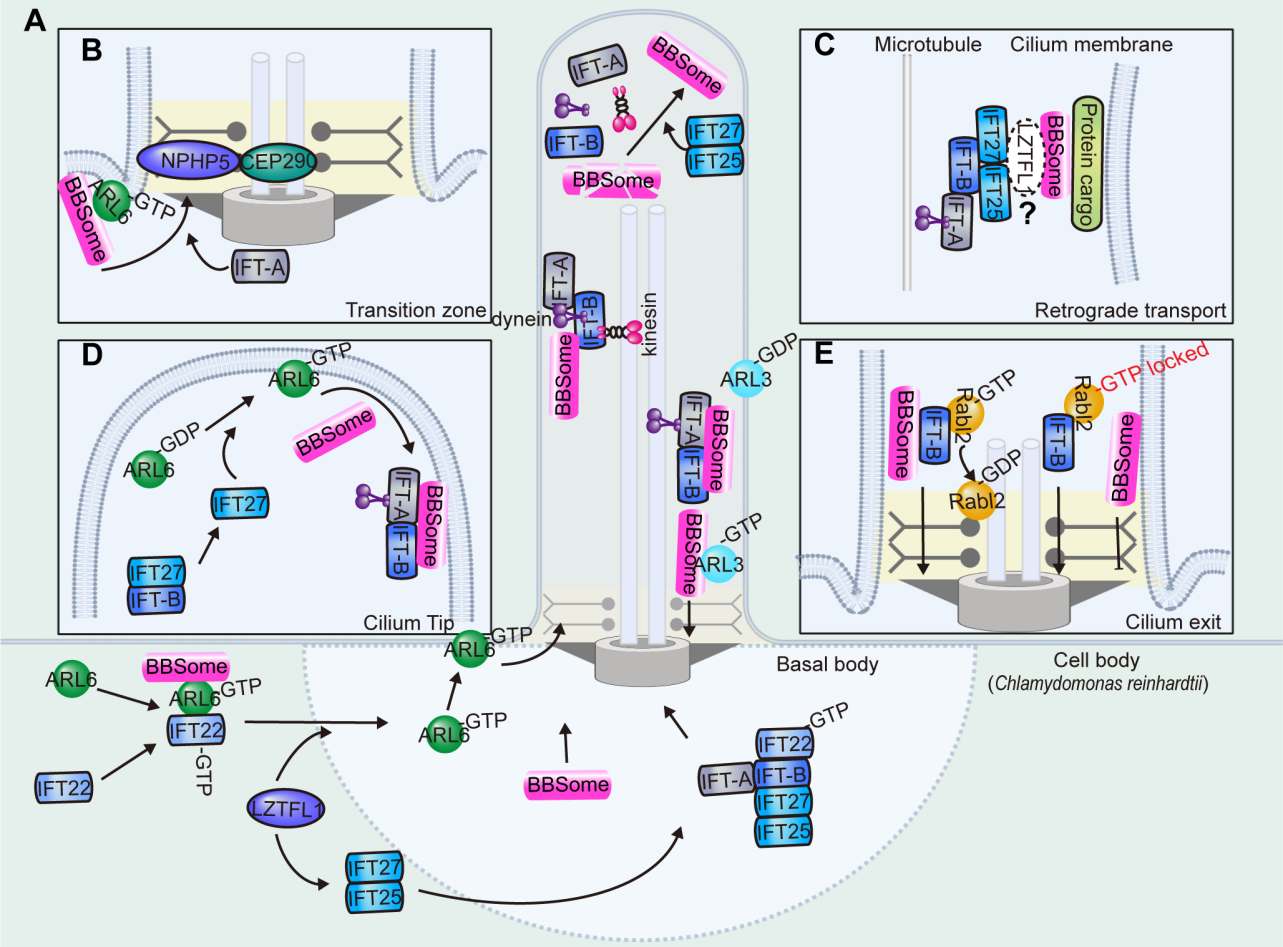
Ciliary transport of the BBSome. (**A**) The BBSome is recruited to the basal body through ARL6-GTP and IFT22-GTP. LZTFL1 also facilitates basal body recruitment of the BBSome. At the ciliary tip, IFT25/27 promotes BBSome reassembly for retrograde transport. At the proximal ciliary region above the transition zone, a portion of cargo-laden BBSome sheds off retrograde IFT and acts as the effector of ARL3 for ciliary retrieval. (**B**) The BBSome is recruited to the membrane as an effector of ARL6-GTP. Ciliary entry of the BBSome is facilitated by IFT-A and transition zone proteins NPHP5 and CEP290. (**C**) The BBSome rides on the retrograde transport train, probably mediated by the IFT-B components IFT27 and IFT25. Whether the adaptor protein LZTFL1 functions as the linker between the BBSome and IFT-B remains to be demonstrated (**D**) At the ciliary tip, IFT27 disassociates from IFT-B to activate ARL6. Then, ARL6-GTP arranges the BBSome onto the membranes for retrograde transport. (**E**) During ciliary exit, the BBSome is transported across the transition zone on IFT-B, where Rabl2-GTP hydrolyses to the Rabl2-GDP form to dissociate from IFT trains. The BBSome sheds off from the IFT-B trains and fails to pass through the transition zone when the IFT-B is persistently bound by the GTP-locked Rabl2.

BBSome components are localized to cilia as well as the basal body and centriolar satellites. As an interdependent entity, deficiency in any BBSome subunit will reduce endogenous levels of the others, suggesting that the BBSome or BBSome intermediates are more stable than the free individual subunits. BBSome or BBSome intermediates are found in the cytoplasm, even when there is no cilium (Prasai et al., 2020). Indeed, the assembled BBSome is proposed to be targeted to the cilium from the basal body, then travel along the cilium *via* anterograde IFT trains. Upon reaching the ciliary tip, the BBSome undergoes a reassembling process to be loaded onto the retrograde IFT trains for ciliary exit (Figure 5A; Liu et al., 2023a; Sun et al., 2021).

### Recruitment to the basal body

The BBSome is present at the basal body in various species, where it is loaded onto IFT trains for cilium entry (Ansley et al., 2003; Lechtreck et al., 2009; Wingfield et al., 2017). BBS1 is particularly abundant at the centrosome in non-ciliated cells and its absence stalls other BBSome components at the centriolar satellites, while the centrosomal localization of BBS1 is not otherwise affected by BBS4 depletion (Prasai et al., 2020). Consistently, BBS5 is absent from the basal bodies in BBS1-depleted *Chlamydomonas* strains (Liu et al., 2021). Thus, BBS1 is predicted to mediate basal body recruitment of the BBSome for ciliary entry (Prasai et al., 2020).

In *C. reinhardtii*, ARL6/IFT22 is required for recruiting the BBSome to the basal body when they are both in GTP-bound states (Figure 5A; Xue et al., 2020). IFT22 is a Rab-like 5 (RABL5) GTPase, and its depletion results in a dramatic decrease in basal body and ciliary BBS1 and BBS5, which are restored by GTP-bound but not GDP-bound IFT22 (Xue et al., 2020). IFT22 binds and stabilizes ARL6 in the cell body independent of their nucleotide states, while their recruitment to the basal body requires both proteins to be in their GTP-bound states (Xue et al., 2020). In contrast with mammalian cells, in which only GTP-ARL6 binds the BBSome, the binding between ARL6 and the BBSome is independent of the nucleotide state of ARL6 in the cell body of *C. reinhardtii*, but only GTP-bound ARL6 can recruit BBSomes to the basal body or bind BBSomes in cilia (Liu et al., 2021). Note that *IFT22* or *ARL6* mutants, in which IFT22 or ARL6 has normal basal body localization but fails to localize in cilia, also retain the ciliary localization of the BBSome, indicating that IFT22 and ARL6 only function in the basal body recruitment of BBSomes but not their ciliary entry (Liu et al., 2021; Xue et al., 2020). Once in the cilium, BBSome transport along the cilium does not require IFT22 or ARL6 either. Thus, IFT22 and ARL6 are only required to recruit the BBSome to the basal body to make it available for loading onto anterograde IFT trains for ciliary entry in *C. reinhardtii*. In addition, *C. reinhardtii* LZTFL1 also directs BBSome basal body recruitment by promoting basal body targeting of ARL6 without binding to IFT22/ARL6 (Sun et al., 2021).

### Ciliary entry and trafficking

In mammalian cells, it is believed that the BBSome is recruited to membrane structures as the major effector of ARL6, which binds the BBSome through BBS1 (Figure 5B; Jin et al., 2010). The ciliary localization of the BBSome and ARL6 are likely interdependent, and both require GTP binding of ARL6. The BBSome subunits BBS1 and BBS18 fail to localize to cilia when ARL6 is depleted in mammalian cells, and ciliary localization of ARL6 dramatically decreases in BBS2-, BBS4-, and BBS5-depleted cells (Jin et al., 2010). However, *C. reinhardtii* ARL6 is not required for the BBSome to enter cilia from the basal body, and it diffuses into the cilium without IFT movement, indicating that the entry of ARL6 to the cilium does not depend on BBSomes either (Liu et al., 2021; Xue et al., 2020). In the retinal photoreceptor of *Arl6* knockout mice, the BBSome is fully assembled and recruited to cilia, indicating that the recruitment of BBSomes to photoreceptor cilia is ARL6 independent (Hsu et al., 2021). These studies suggest that the ciliary entry of the BBSome is differentially regulated across tissues or species, and the detailed mechanisms underlying its ciliary entry are still not well characterized.

It is known that only the fully assembled BBSome holo-complex can gain entry into the ciliary compartment, as the loss of any single subunit prevents its ciliary trafficking (Seo et al., 2011; Lechtreck et al., 2009). However, in the absence of BBS2 or BBS7, a subcomplex (BBS1/5/8/9) was able to enter retinal photoreceptor cilia, providing additional evidence that the residual BBSome, reminiscent of the partial BBSome in *D. melanogaster*, enters cilia (Hsu et al., 2021). Ciliary entry requires passage of the BBSome through a special region at the base of the cilium, known as the transition zone. The transition zone acts as a permeability barrier to control the entry and exit of ciliary proteins (Park and Leroux, 2022). It has been reported that the transition zone protein NPHP5 interacts with the BBSome to mediate its integrity (Barbelanne et al., 2015). Depletion of NPHP5 or expression of *NPHP5* mutants defective in BBSome binding leads to dissociation of BBS2 and BBS5 from the BBSome and loss of ciliary BBS2 and BBS5, while other BBSome subunits enter cilia normally (Barbelanne et al., 2015). CEP290, another transition zone protein that directly binds NPHP5 (Barbelanne et al., 2013; Sang et al., 2011; Schäfer et al., 2008), can also regulate BBSome integrity. The absence of CEP290 abolishes the ciliary localization of BBS2, BBS5, and BBS8 without affecting the ciliary localization of BBS1, BBS7, BBS9, and BBS18. Whether the ciliary targeting of BBS4 is impaired by CEP290 depletion is still arguable (Barbelanne et al., 2015; Stowe et al., 2012). Considering that the BBSome is missing in some subunits instead of completely compromised for ciliary entry in the absence of NPHP5 or Cep290 (Barbelanne et al., 2013), transition zone-localized NPHP5 and CEP290 potentially accomplish two goals (Figure 5B): regulating BBSome integrity and forming a diffusion barrier that allows the selective passage of the holo-BBSome complex into the cilium. Of note, the loss of CEP290 in *C. reinhardtii* increases the amount of BBS4 and IFT-B complex proteins in the flagellum (Craige et al., 2010). Thus, the precise mechanisms underlying ciliary entry control still deserve further exploration.

Recent studies have revealed the function of IFT-A in ciliary entry, and the BBSome is abnormally localized in cells defective in IFT-A subunits IFT139 (non-core subunit), IFT144 (core subunit), and the IFT-A interactor C11ORF74 (Takahara et al., 2019; Hirano et al., 2017). BBS9 tends to accumulate within cilia, particularly at the distal tip in IFT139-depleted cells, but it is absent from cilia in IFT144- or C11ORF74-depleted cells, representing a functional difference in IFT-A core and non-core subunits.

**Figure 6.**
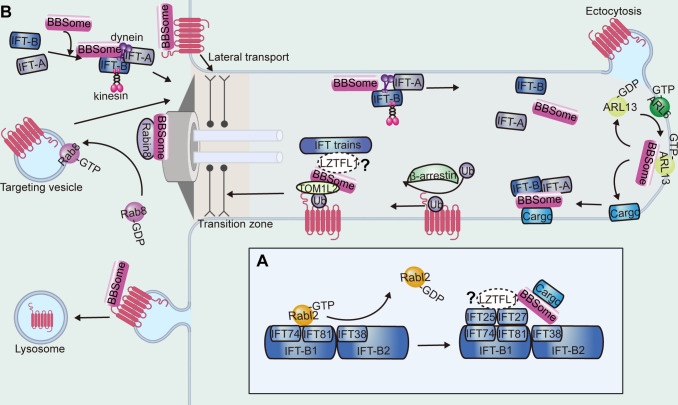
Functions of the BBSome in the cilium. (**A**) The anchoring of the BBSome on IFT trains. The BBSome interacts with IFT-B through IFT38. The Rabl2 GTPase and the IFT25-IFT27 dimer bind to IFT74-IFT81 of IFT-B in a mutually exclusive manner and may regulate BBSome-mediated cargo loading. (**B**) During the cargo’s ciliary entry, the BBSome either regulates vesicle targeting in a Rab8-Rabin8-dependent manner or regulates the cargo’s lateral transport between the plasma and ciliary membrane. At the ciliary base and at the ciliary tip, the BBSome may regulate IFT assembly to ensure their ciliary entry and turnaround and hold them together during transport. At the ciliary tip, ARL6 and ARL13 regulate cargo pickup by the BBSome for retrieval. During the cargo’s exit, β-arrestin arranges the cargo for ubiquitin modification, followed by BBSome-mediated exit across the transition zone. TOM1L2 may function as an adapter between the BBSome and ubiquitin sidechains. The BBSome facilitates endocytic sorting of select membrane proteins at the base of the cilium. The unretrieved GPCRs can also be shed into ectocytosis vesicles for disposal.

Inside the cilium, the BBSome moves both anterogradely and retrogradely at the same rates as IFT (Lechtreck et al., 2009; Ou et al., 2005). It is thought that the BBSome is carried by IFT but is an adapter rather than an integral component of the IFT machinery. In the cilia of mammalian olfactory sensory neurons, the BBSome components undergo bidirectional particle movement with similar velocities to IFT proteins and participate in IFT as a constituent in 1:1 stoichiometry, in contrast to BBSome proteins only associating with a subset of IFT particles in unstimulated IMCD3 cells and *C. reinhardtii* (Lechtreck et al., 2009; Williams et al., 2014; Ye et al., 2018). The docking of BBSomes to the IFT-B complex is predicted to involve IFT38, which interacts with BBS1-BBS2-BBS9 through the C-terminal tail and is essential for retrograde transport of the BBSome (Nozaki et al., 2019; Wang et al., 2022). Structurally, a positive patch on a region of BBS9 that interacts with BBS1 and BBS2 constitutes the ideal candidate for binding to IFT38 (Yang et al., 2020). Other components from IFT-B, such as IFT74-IFT81 and IFT25-IFT27, may also contribute to BBSome-dependent transport (Zhou et al., 2022b; Luo et al., 2021). IFT74-IFT81 interacts with IFT25-IFT27, which can be competitively regulated by Rabl2 GTPase, and this interaction is important for BBSome-associated ciliary defects (Figure 6A; Luo et al., 2021; Zhou et al., 2022a). The detailed mechanisms of BBSome trafficking on the IFT trains still need further investigation.

### Turnaround at the ciliary tip

Once reaching the ciliary tip, the BBSome and IFT trains are thought to undergo remodeling before turning around for retrograde transport to exit cilia (Figure 5A; Wei et al., 2012; Wingfield et al., 2021). IFT27, a small G protein with very low intrinsic GTPase activity distantly related to the Rab subfamily of Ras-like GTPases, is a component of IFT-B and forms a stable heterodimer with IFT25 (Bhogaraju et al., 2011). BBSome subunits (BBS5 and BBS9) and BBS regulators (ARL6 and LZTFL1) accumulate in the cilia of *IFT27*-knockout cells (Eguether et al., 2014). The ciliary exit rate of the BBSome measured by fluorescence loss after photobleaching (FLAP) decreases in *IFT27*-knockdown cells, while ciliary entry measured by fluorescence recovery after photobleaching (FRAP) does not alter, indicating that a defect in ciliary exit but not entry contributes to ciliary accumulation of the BBSome (Liew et al., 2014). In *LZTFL1*-mutant cells, ciliary staining of IFT27 remains normal, suggesting that LZTFL1 functions downstream of IFT27 (Eguether et al., 2014). BBSome depletion does not affect IFT27 and LZTFL1 localization, indicating that they function upstream of the BBSome (Eguether et al., 2014). Based on the above observations, the proposed model indicates that LZTFL1 coordinates interactions between the BBSome and IFT particles to remove ciliary cargoes from the cilium (Figure 5C). However, *Chlamydomonas* LZTFL1 does not interact with IFT in cilia, thus refuting the role of LZTFL1 as the BBSome/IFT linker, at least in *C. reinhardtii* (Sun et al., 2021).

In an alternative model, IFT27/25 may regulate BBSome loading onto retrograde IFT for ciliary removal at the ciliary tip. Mammalian IFT27 was previously considered to work as the guanine exchange factor (GEF) for ARL6 at the ciliary tip to prepare for retrograde BBSome trafficking (Figure 5D; Liew et al., 2014). In murine cells, IFT25/IFT27 is transiently released from IFT-B at the distal tip of the cilium, where IFT27 then binds and stabilizes the nucleotide-free form of ARL6 to promote ARL6 activation and subsequent capture of the BBSome to membranes (Liew et al., 2014). However, ARL6 is not required for loading BBSomes onto retrograde IFT trains for ciliary exit at the ciliary tip in *C. reinhardtii*, as ARL6 and BBSome transport separately in *C. reinhardtii* cilia and ciliary loss of ARL6 does not cause ciliary hyperaccumulation of BBS1, BBS4, BBS5, or BBS7 (Liu et al., 2021). The ARL6 GEF activity of IFT27 should also be considered with caution, considering that the GEF activity of IFT27 *in vitro* is low and the biochemical assays are performed on whole cell samples rather than cilia alone (Liew et al., 2014). In *C. reinhardtii*, IFT25/27 does not depend on IFT27’s nucleotide state to cycle on and off IFT-B and fails to activate ARL6 *in vitro*, arguing against a GEF function for IFT27 (Liu et al., 2023a; Liu et al., 2023b). Similar to IFT27, IFT25 depletion also has no effect on ciliary entry of the BBSome, but it does impair BBSome ciliary exit (Dong et al., 2017). Closely related to IFT27, IFT25 depletion causes a dramatic decrease in IFT27 levels and a BBSome exit defect similar to the defects induced by IFT27 depletion both in mice and *C. reinhardtii* (Eguether et al., 2014; Dong et al., 2017; Keady et al., 2012). At the ciliary tip of *IFT25*-knockdown cells, BBS1 and BBS5 cannot assemble into the intact BBSome and hyperaccumulate there (Liu et al., 2021). In summary, *Chlamydomonas* IFT25/27 promotes BBSome reassembly for coupling with IFT-B during the BBSome turnaround at the ciliary tip (Figure 5A; Sun et al., 2021; Dong et al., 2017). Meanwhile, *Chlamydomonas* LZTFL1 mediates BBSome reassembly at the ciliary tip for its removal by stabilizing IFT25/27 in the cell body (Sun et al., 2021). Note that *Ift25*, *Ift27*, and *Lztfl1* homologs are absent from the genomes of *D. melanogaster* and *C. elegans* (van Dam et al., 2013), suggesting a different mechanism underlying retrograde BBSome transport in flies and worms.

### Ciliary removal

For ciliary exit, the BBSome must pass through the transition zone (Ye et al., 2018). Rabl2 is a Rab-like small GTPase capable of self-activation into a GTP-binding form, and it was previously found to initiate ciliary entry of IFT (Kanie et al., 2017). BBS5, BBS7, and ARL6 accumulate in cilia in GTP-locked *Rabl2* mutant (Q80L) cells (Duan et al., 2021): although anterograde and retrograde transport velocities were not affected, BBSome export dramatically decreased. In cells expressing Rabl2-Q80L, super-resolution imaging revealed that the BBSome distributes exclusively above the transition zone, where the BBSome reaches but fails to pass across. Interestingly, disrupting the Rabl2-Q80L and IFT-B interaction ensures transition zone passage of the BBSome, indicating that persistent binding of Rabl2-GTP to IFT-B results in shedding of the BBSome before it passes across the transition zone (Duan et al., 2021). Thus, it is proposed that the BBSome is transported and reaches the transition zone on the IFT-B retrograde machinery in the presence of Rabl2-GTP, which hydrolyses to its Rabl2-GDP form to dissociate from IFT trains and to allow the BBSome to pass through the transition zone (Figure 5E). In *C. reinhardtii*, a portion of cargo-laden BBSome sheds off retrograde IFT at the proximal ciliary region and acts as the effector of ARL3 to pass the transition zone for ciliary retrieval, likely *via* diffusion (Figure 5A; Liu et al., 2022). Whether a Rabl2-ARL3 cascade exists, in which Rabl2 functions as the GEF of ARL3, remains to be confirmed in *C. reinhardtii*.

## Ciliary mechanisms and functions of the BBSome

Unlike IFT mutants, which typically result in pronounced structural ciliary abnormalities, BBSome mutants have varied effects on cilium assembly and in most cases are dispensable for ciliogenesis (Loktev et al., 2008; Hey et al., 2021). Knockout of BBSome components in mice does not abolish ciliogenesis globally; however, there is evidence of cilia assembly defects in BBS-deficient cells, such as the absence of sperm flagella and malformed photoreceptor outer segments (Hsu et al., 2017; Nishimura et al., 2004; Davis et al., 2007; Zhang et al., 2013; Mykytyn et al., 2004). Meanwhile, ciliary membrane composition is affected by the absence of the BBSome, indicating a role for them in ciliary transport.

Early studies revealed that the BBSome is required for ciliary entry of several GPCRs, as somatostatin receptor type 3 (SSTR3) and melanin-concentrating hormone receptor 1 (MCHR1) are absent from hippocampal neuron cilia in *Bbs2*- or *Bbs4*-mutant mice (Berbari et al., 2008b). NPY2R failed to localize to cilia in *Bbs18*-mutant mice hypothalamus (Loktev and Jackson, 2013). However, recent results support a role for the BBSome in ciliary exit, excluding non-ciliary proteins leaked into the cilium, and regulating the balance of ciliary receptor concentration (Lechtreck et al., 2009; Ye et al., 2018). For example, SSTR3 was also shown to exit the cilium in IMCD3 cells in a BBSome-dependent manner (Ye et al., 2018). These pleiotropic outcomes may be influenced by the activation state of the receptors or be cell type- and species-dependent, and the detailed mechanisms remain unclear (Domire et al., 2011). In summary, the BBSome acts as a cargo adaptor of the IFT, expanding the cargo range of IFT in ciliary trafficking to regulate the movement of cargo proteins in and out of cilia (Lechtreck, 2022).

### Target recognition

To mediate cargo transport, the BBSome should be able to bind cargoes on specific domains involved in cilium targeting (ciliary targeting sequence, CTS). It has been reported that the third intracellular loops of SSTR3, 5-hydroxytryptamine receptor 6 (HTR6), MCHR1, and dopamine receptor 1 (D1) are sufficient for ciliary localization and can be bound by the holo-BBSome (Jin et al., 2010; Berbari et al., 2008a; Domire et al., 2011). Comparing these loops reveals a loose consensus sequence, Ax[S/A]xQ (Berbari et al., 2008a). Other CTSs include the C-terminal VxP sequence in rhodopsin, polycystin 1, and polycystin 2 (Deretic et al., 1998; Su et al., 2015) and the (F/Y/W)(K/R) motif in Smo (Corbit et al., 2005). There is also some evidence implicating the RVxP motif in the N-terminal domain of polycystin 2 (Geng et al., 2006). However, a recent study challenged the legitimacy of some of the previously identified hot spots, and showed that the heterologously expressed BBSome hexamer (BBS1, 4, 5, 8, 9, and 18) has robust affinity for the (F/Y/W)(K/R) motif and other sequences containing one or more basic or aromatic residues (Klink et al., 2017). Note that these CTSs seem to be sufficient but not necessary for ciliary cargo localization, indicating that the regulation of cargo localization is rather complex and may involve several parallel mechanisms.

BBS1 is considered the major subunit for BBSome cargo recognition, interacting with several ciliary cargoes including Smo, Patched, leptin receptor, and polycystin 1 (Zhang et al., 2012a; Seo et al., 2009; Su et al., 2014). Besides BBS1, the C-terminal of Smo is also reported to bind the BBSome through BBS4, 5, and 7 (Seo et al., 2011). BBS5 specifically interacts with the third intracellular loop of D1 (Domire et al., 2011). Regardless, cargo binding may depend on the 3-dimensional arrangement of the holo-BBSome, which exploits different binding modes to cope with different cargoes (Singh et al., 2020).

### Cargo entry

BBSome-mediated ciliary entry can be achieved by targeting vesicles to the cilium to promote ciliary membrane elongation or by functioning as a planar coat for lateral transport between the plasma and ciliary membranes (Figure 6B; Jin et al., 2010; Nachury et al., 2007). At the basal body, the BBSome interacts with Rabin8, a guanine nucleotide exchange factor (GEF) for Rab8, through BBS1, which may potentiate the GEF activity of Rabin8 to facilitate vesicle docking and fusion to the base of the cilium (Nachury et al., 2007). Interestingly, a recent study also established a unique role for the BBSome in preventing entry (Yu et al., 2020). Fluorescence protein-tagged carbonic anhydrase 6 (CAH6-mNG) preferentially localized to the trans-flagellum emerging from the older basal body, which was established early during flagellar assembly and restored after photobleaching. The uneven distribution in both cilia was disrupted in *BBS1*-mutant strains. Considering that the BBSome localizes to the transition zone, it may serve as a roadblock that prevents the entry of CAH6-mNG into the cis-flagellum (Dean et al., 2016; Yu et al., 2020).

### Cargo exit

It has been reported that several GPCRs, such as Smo, GPR161, Patched, and D1, accumulate in BBS-mutant cilia (Domire et al., 2011; Nozaki et al., 2018). A role for the BBSome in ciliary exit is further supported by a proteomic analysis that detected over 130 non-ciliary proteins accumulated in the photoreceptor outer segments of *Bbs*-mutant mice (Datta et al., 2015). It has been suggested that the BBSome promotes ciliary export of proteins by linking them to IFT particles for retrograde transport, facilitating their passage across the transition zone, and shedding ciliary extracellular vesicles for disposal (Figure 6B).

The interplay between the BBSome and IFT can be divided into three major events: movement on IFT as a cargo adaptor, maintaining IFT integrity, and regulating IFT assembly at the ciliary base for entry and at the ciliary tip for turnaround (Figure 6B). In *C. reinhardtii*, single particle imaging revealed that the IFT movement of phospholipase D1 (PLD) was abolished in *BBS4* mutants, resulting in PLD accumulation inside cilia (Liu and Lechtreck, 2018). This accumulation was abolished by reintroducing the wild-type BBSome into *BBS*-mutant strains, which caused rapid removal of PLD from the mutant cilia (Lechtreck et al., 2013). PLD travels predominately on the IFT trains together with the BBSome, indicating that the BBSome is a cargo adapter ensuring the ciliary export of PLD on IFT trains (Liu and Lechtreck, 2018). In brief, the BBSome moves in association with IFT particles to mediate the ciliary exit and the transition zone passage of membrane proteins by connecting them with the IFT-B complex (Lechtreck et al., 2009; Nozaki et al., 2019).

The BBSome plays a role in maintaining IFT integrity and organization, holding IFT-A and IFT-B together. IFT-A and IFT-B are observed to move together in *C. elegans* sensory cilia, while they separate to move at different speeds by the heterotrimeric kinesin-2 and homodimeric OSM-3 motors, respectively, in *BBS7* and *BBS8* mutants (Ou et al., 2005). Consistently, asynchronous rates of IFT-A/B particle movements were observed within the olfactory sensory neuron cilia of *Bbs4*-mutant mice (Uytingco et al., 2019). However, this function is species-specific, as BBS proteins seem to be dispensable for IFT assembly and transport in *C. reinhardtii* (Lechtreck et al., 2009).

In *C. elegans*, it has also been shown that the BBSome is the key player in regulating IFT assembly and turnaround in cilia (Figure 6B; Wei et al., 2012). The ciliary localization and motility of IFT proteins OSM-5, CHE-11, and CHE-2 are altered in *BBS7-* and *BBS8*-mutant *C. elegans* strains, indicating that the *C. elegans* BBSome may facilitate the selective assembly of IFT protein components into the IFT particle at the basal body before its proper movement inside cilia (Wei et al., 2012). At the ciliary tip, IFT particles undergo a dissociation and reassembly process, while the absence of the *C. elegans* BBSome leads to defective reassembly of the IFT complex and the specific accumulation of IFT-B components at the ciliary tip (Wei et al., 2012).

In mammalian cells, retrograde trafficking and/or export of ciliary GPCRs are impaired in the absence of ARL6, and PLD is observed to accumulate in the ciliary tip of *Chlamydomonas* mutant that lacks ciliary ARL6 (Liu et al., 2021; Liew et al., 2014; Zhang et al., 2011). At the ciliary tip, GTP-bound *Chlamydomonas* ARL6 is required for recruiting BBSomes to the ciliary membrane, making the BBSome available for picking up ciliary signaling cargoes (Figure 6B; Liu et al., 2021). Recent studies have shown that the ARL6-BBS1 interaction is reinforced by BBS9 (Nozaki et al., 2018). Ciliary GPCR trafficking defects in BBS1-depleted cells cannot be rescued by BBS1 mutants lacking BBS9-binding ability, indicating that intact BBSomes are required for retrograde trafficking of GPCRs out of cilia (Nozaki et al., 2018). Besides ARL6, ARL13 is also involved in BBSome-dependent cargo transport in *Chlamydomonas* (Liu et al., 2023a; Dai et al., 2022). In the *Chlamydomonas arl13* mutant, a set of membrane-associated proteins accumulated in cilia, although the IFT movement of BBSomes was mostly normal, suggesting that ARL13 is probably required for ensuring that cargo proteins can be picked up by the BBSome carriers but not for maintaining BBSome IFT integrity (Dai et al., 2022). At the ciliary tip, ARL13 is activated by ARL6 and recruits the BBSome as an ARL13 effector to anchor to the ciliary membrane, making it spatiotemporally available for coupling with cargoes (Figure 6B; Liu et al., 2023a). Regardless, the detail of how the BBSome is organized into a complex that can recognize signaling molecules in the ciliary membrane for IFT needs further exploration.

Facilitated by the BBSome, ciliary exit is supposed to be a two-step process in which activated GPCRs pass through the transition zone before crossing a periciliary barrier (Ye et al., 2018). It has been reported that activation of ciliary GPCRs such as Smo and SSTR3 drives the ARL6-dependent assembly of large, highly processive, and cargo-laden retrograde BBSome trains. Single-molecule imaging revealed that the assembly of BBSome trains enables the lateral transport of ciliary GPCRs across the transition zone but not a second periciliary diffusion barrier (Ye et al., 2018).

Ciliary GPCRs can also be retrieved by shedding ciliary extracellular vesicles and removal from the periciliary membrane compartment (Figure 6B). It has been reported that GPCRs failing to undergo BBSome-mediated retrieval concentrate into membranous bud at the tips of cilia and undergo ectocytosis to compensate for BBSome-related ciliary removal defects (Nager et al., 2017). Interestingly, the BBSome negatively regulates extracellular vesicle release by targeting RAB28, and possibly also other extracellular vesicle regulators, to cilia (Akella et al., 2020; Jensen et al., 2016). Note that both a lack of ciliary localization in BBSome-defective cells and BBSome-dependent ciliary exit have been reported for SSTR3 in different cell types (Berbari et al., 2008a; Berbari et al., 2008b; Ye et al., 2018; Nager et al., 2017). Considering that the redundant GPCRs can be removed by ectocytosis in BBSome-defective cells, thus leaving cilia devoid of the protein, this disparity can be reasonably explained (Nager et al., 2017). Furthermore, it has been reported that BBS4 and BBS5 have redundant functions in the degradative sorting of ciliary sensory receptors (Xu et al., 2015). The BBSome is reported to facilitate endocytic sorting of select membrane proteins at the cilium base (Langousis et al., 2016). As the exported ciliary membrane proteins undergo clathrin-mediated endocytosis outside of the ciliary compartment for their clearance, the involvement of the BBSome in periciliary membrane dynamics still needs to be explored (Pal et al., 2016).

Questions also remain as how the BBSome distinguishes cargoes – such as active and inactive GPCR states – for export, and there must exist another layer of regulation that commits activated GPCRs for exit. Recent studies have shown that a common mechanism of ciliary removal of activated GPCRs is ubiquitination by K63 linkages for BBSome-dependent exit (Figure 6B; Shinde et al., 2020). In *C. elegans*, attachment of ubiquitin (Ub) to the cytoplasmic end of polycystin 2 results in its ciliary clearance (Xu et al., 2015; Hu et al., 2007). It has also been reported that ubiquitinated proteins accumulate in the cilia of BBSome-defective mammalian cells, suggesting that the BBSome might sort ubiquitinated signaling receptors out of cilia (Shinde et al., 2020). Even though Ub copurifies with the BBSome in *T. brucei*, suggesting a potential interaction between BBSomes and Ub chains, the direct biochemical evidence for Ub chain binding is still missing (Langousis et al., 2016). Anyway, an interaction between BBSomes and Ub chains may enhance BBSome-cargo interactions to enable sorting of ubiquitinated GPCRs out of cilia. Alternatively, adaptor proteins, such as target of Myb1 like 2 membrane trafficking protein (TOM1L2), which bridges the BBSome to its ubiquitinated cargoes, might be involved (Shinde et al., 2023). Upon activation, an intermediate protein, β-arrestin, may well first arrange the activated GPCRs for Ub linkage, followed by BBSome-mediated retrieval (Shinde et al., 2020). Ubiquitinated Smo is retained in IFT27-, LZTFL1-, and BBS2-depleted cells, suggesting an IFT-dependent removal mechanism, in which the BBSome binds both Ub and Smo and couples with the IFT for ciliary removal (Desai et al., 2020). Different from Smo, ubiquitinated SSTR3 does not accumulate in IFT27-depleted cells, indicating that an IFT-independent alternative mechanism exists. Thus, different GPCRs may rely on different mechanisms for ciliary removal.

## Conclusions and perspectives

There has been significant progress in establishing the biological significance of the BBSome over the last decade, not least confirming its fundamental roles in regulating ciliary dynamics and non-ciliary processes. However, several questions about the BBSome remain. How is the BBSome spatiotemporally assembled and transported in cilia? How do BBSome subunits cooperate and function in selective ciliary and non-ciliary events? How are the functions of the BBSome mechanistically associated with symptoms? Bolstered by significant advances in modern technologies such as super-resolution microscopy, time-lapse and single molecular imaging, and cryo-electron microscopy, future studies are likely to further extend our understanding of BBSome regulation and related diseases, providing novel targets for clinical treatment and drug development.

Characterized by a high degree of genetic and clinical heterogeneity, there is no cure for BBS, and these patients are treated symptomatically with multidisciplinary approaches (Caba et al., 2022). Molecular genetic testing is recommended to help focus on particular pathogenic genes and provide the possibility of gene therapy. Indeed, there have been several successful attempts at gene therapy in BBS mouse models, at least for eye pathology, since the eyes are relatively more accessible than other organs for gene delivery. In *Bbs1*- and *Bbs4*-mutant mice, subretinal delivery of adeno-associated virus (AAV)-mediated BBS proteins ameliorated rhodopsin mislocalization and retinal degeneration (Seo et al., 2013; Simons et al., 2011). A U1 small nuclear RNA (U1)-mediated therapeutic approach corrected the splice defect in *BBS1*-variant patient-derived fibroblasts *in vitro*, suggesting a potential gene therapy to overcome the pathogenic effects of splice donor site mutations (Schmid et al., 2011). In another study, translational readthrough-inducing drugs were used to restore full-length protein, bypassing in-frame premature termination codons in *BBS2* variant patient fibroblasts, highlighting the feasibility of the approach in nonsense mutation-dependent ciliopathies (Eintracht et al., 2021). A recent study reported that an adenovirus-delivered wild-type *Bbs1* gene could restore ciliation and acute odor responses in olfactory sensory neurons in *Bbs1*-mutant mice, supporting the viability of gene therapy in congenital olfactory disorders in BBS patients (Williams et al., 2017). Aided by advanced diagnostic techniques, detailed phenotyping, and pharmacogenomic analysis, more BBS patients are likely to benefit from personalized medicine and gene therapy in the future.
